# Occurrence, Migration Behavior, and Environmental Burden of Phthalate Esters in Flooring Materials Used in Newly Renovated Chinese Homes

**DOI:** 10.3390/toxics13070517

**Published:** 2025-06-20

**Authors:** Ying Zhang, Li-Bo Chen, Hao-Yang Shen, Zi-Chao Wu, Ning-Zheng Zhu, Chong-Jing Gao, Ying Guo

**Affiliations:** 1 Zhejiang Provincial Top Discipline of Biological Engineering (Level A), Zhejiang Wanli University, Ningbo 315100, China; z_yingzy@163.com (Y.Z.); lbchen0107@163.com (L.-B.C.); shenhaoy2023@163.com (H.-Y.S.); wzc4811@163.com (Z.-C.W.); 2 State Key Laboratory of Pollution Control and Resources Reuse, College of Environmental Science and Engineering, Tongji University, Shanghai 200092, China; zhuningzheng1984@163.com; 3 Guangdong Key Laboratory of Environmental Pollution and Health, College of Environment and Climate, Jinan University, Guangzhou 510632, China; yingguo2004@jnu.edu.cn

**Keywords:** phthalates, flooring, migration, environmental burden

## Abstract

Phthalic acid esters (PAEs), a class of synthetic semi-volatile organic compounds, are extensively incorporated into decorative materials. However, their specific occurrence, migration behaviors, and environmental impact on these materials—which comprise the largest surface areas in residential settings—remain insufficiently understood. This study investigated the distribution, emission dynamics, and environmental burdens of PAEs in flooring commonly used in Chinese households. The results showed that PAEs are widespread in flooring, with total concentrations ranging from 1220 to 166,000 ng/g (14,100 ng/g, median value). Solid wood flooring (55,900 ng/g) exhibited significantly higher PAE levels compared to engineered flooring (22,600 ng/g) and laminate flooring (4000 ng/g) (*p* < 0.05). Migration experiments revealed that solid wood flooring tended to continuously release PAEs, laminate flooring showed a pronounced capacity for PAE absorption, and engineered flooring exhibited both release and absorption behaviors. The initial PAE concentration is the dominant factor influencing migration rates, while the flooring type and substrate density also contribute to varying degrees. The estimated environmental burdens of PAEs resulting from flooring in newly renovated Chinese households ranged from 3.63 × 10^9^ ng to 3.45 × 10^11^ ng, with a median value of 1.23 × 10^10^ ng. Households in the eastern and southwestern regions exhibited the highest PAE burdens, while the southern region showed the lowest. Socioeconomic factors such as residential floor area, number of rooms, household income, and renovation budget significantly influenced the environmental burden of PAEs derived from flooring.

## 1. Introduction

Modern home decoration is increasingly characterized by high levels of integration and material complexity, with both the variety and quantity of decorative materials rising significantly. Compared to traditional materials, modern materials exhibit a greater capacity to release harmful substances [1,2], notably through the persistent release and redistribution of semi-volatile organic compounds (SVOCs) from decorative materials, leading to the phenomenon known as third-hand pollution [3]. This refers to the persistent presence and accumulation of pollutants on indoor surfaces, dust, or porous materials even after the primary pollution sources have been removed [4]. Particularly noteworthy is the case of wood-based materials—long regarded as relatively safe—which have recently been identified as exhibiting dual functions, acting both as sources of emissions and as secondary sinks for indoor pollutants [5]. Changes in ambient conditions such as temperature and ventilation can trigger not only the emission of SVOCs from these materials but also their reabsorption from the surrounding environment [6]. This behavior challenges the conventional assumption that removing pollution sources is sufficient to eliminate associated risks.

As the most widely produced plasticizers, phthalic acid esters (PAEs) have become increasingly prominent indoor pollutants. Due to their excellent plasticizing properties, PAEs are widely used in building materials, furniture boards, and various household products [7,8]. However, since PAEs are not chemically bonded to the polymers to which they are added, they can easily escape from the materials into the air and subsequently migrate into the surrounding environment or other porous, adsorptive surfaces [9,10]. This characteristic has made PAEs one of the most abundant organic pollutants found in indoor air and dust. Indeed, studies have shown that the concentrations of PAEs in indoor environments, particularly in household dust, are at concentrations that are several orders of magnitude higher than those of other chemical compounds [11]. Moreover, a combination of factors such as the extensive use of synthetic materials, limited ventilation, and prolonged indoor occupancy contributes to a situation where indoor air pollution by PAEs is often significantly more severe than that in outdoor environments [12,13].

Wood-based materials are a significant indoor source of PAEs [14,15,16,17]. Previous research has demonstrated that even after long-term use, wood-based materials can continue to release or reabsorb PAEs, posing persistent threats to indoor environments and human health [18]. The health risks associated with PAEs are well-documented and alarming. Chronic exposure to PAEs has been linked to a variety of adverse effects, including endocrine disruption [19,20,21], reproductive abnormalities [22,23,24,25], developmental delays [26,27,28,29], and metabolic disorders [30,31]. However, current research on the migration of PAEs in wood-based decorative materials remains limited. Most experimental studies on PAE migration rely on short-term accelerated aging tests, often overlooking the bidirectional mass transfer between materials and the environment. This leads to systematic biases in estimating emission rates from materials. Furthermore, the inherent heterogeneity of wood substrates has not been thoroughly investigated—key parameters such as the initial concentration of pollutants, density, and surface treatment are frequently simplified into single variables [32].

This study aims to investigate the occurrence and migration behaviors of PAEs in wood-based decorative materials and indoor PAE pollution. On this basis, it further analyzes the relationship between material properties and PAE migration, identifies the key factors influencing PAE release, and assesses the impact of wood-based materials on indoor PAE pollution. The findings will not only help guide the selection of safer and more sustainable decorative materials but also provide a scientific basis for formulating policies to reduce indoor PAE contamination and protect public health.

## 2. Materials and Methods

### 2.1. Chemicals and Reagents

Nine target PAE compounds, including dimethyl phthalate (DMP), diethyl phthalate (DEP), di-iso-butyl phthalate (DIBP), di-n-butyl phthalate (DBP), di-hexyl phthalate (DHxP), butyl-benzyl phthalate (BBP), di-cyclohexyl phthalate (DCHP), bis(2-ethylhexyl) phthalate (DEHP), and di-n-octyl phthalate (DOP), along with their isotope-labeled internal standards (DMP-d_4_, DEP-d_4_, DIBP-d_4_, DBP-d_4_, DHxP-d_4_, BBP-d_4_, DCHP-d_4_, DEHP-d_4_, and DOP-d_4_), were purchased from AccuStandard (New Haven, CT, USA) and C/D/N Isotopes (Pointe-Claire, QC, Canada). The organic solvents used for analyte extraction and standard solution preparation were methanol and hexane, both of which were of LC-grade and had a purity of ≥98%. They were purchased from Thermo Fisher Scientific Co., Ltd. (Shanghai, China). LC-grade ultrapure water was also sourced from Thermo Fisher Scientific Co., Ltd. (Shanghai, China).

### 2.2. Questionnaire and Sample Collection

To understand the use of decoration materials in newly renovated homes in China, an online questionnaire survey was conducted (Table S1). The survey collected information on the basic household details and the use of decoration materials, including demographic information (gender, age, height, and weight of family members), geographical information (city of residence, urban/rural attributes), housing characteristics (housing type, living area, number of rooms), time spent at home, household income, renovation time, renovation budget, use of decoration materials (material types, quantity), and frequency of ventilation. After excluding invalid questionnaires due to incomplete responses, logical inconsistencies, or duplicate submissions, 675 valid responses were obtained, covering all provinces, autonomous regions, and municipalities in China, except for Taiwan Province and the Hong Kong Special Administrative Region.

Based on the results of the questionnaire survey in Table S1, a total of 42 flooring samples commonly used by Chinese residents were collected from both decoration material stores and online shops. The flooring types primarily included solid wood flooring (*n* = 13), engineered wood flooring (*n* = 15), and laminate wood flooring (*n* = 14). All the flooring samples were sourced from 21 popular brands commonly used by Chinese residents. Detailed information on the flooring is shown in Table S2 in Supplementary Materials. During sample collection, a sample information database was established to record detailed information about each sample, including flooring type, origin, material, and processing technology.

### 2.3. PAEs Migration Experiment

From January 2022 to January 2023, a PAE migration experiment was conducted on wood-based flooring. To simulate real-life usage conditions, a 30 m^2^ room with good lighting and ventilation, which had been renovated over 15 years ago, was selected for the experiment. To ensure stable environmental conditions, the temperature in the room was maintained between 20 and 22 °C, and the humidity was kept at 45–50% throughout the experiment. The three types of wood-based flooring samples were removed from their surface packaging materials. Each sample was cut into small pieces (10 cm × 10 cm) and placed on clean stainless steel plates, spaced more than 50 cm apart, and separated by stainless steel dividers to prevent cross-contamination between samples. To avoid interference from any residual PAEs on the surface of the samples and cutting tools, the sample surfaces, milling tool heads, and saw blades were wiped with hexane before cutting. During the experiment, the temperature, humidity, and air flow speed in the room were continuously monitored and recorded to ensure the controllability of the experimental conditions and the reliability of the data.

### 2.4. Sample Preparation and Instrumental Analysis

On the 1st and 365th days of the release experiment, a floor powder sample was collected from each flooring sample. First, the surface of each flooring sample was cleaned with a pre-cleaned Kimwipe to remove PAEs on the surface of the sample to remove any dust and airborne contaminants. Then, the floor powder was scraped from different areas of the sample using an electric grinder cleaned with hexane. This procedure removed material from both the surface and subsurface layers of the flooring. For each flooring sample, approximately 1.0 g of powder was ground, and then the powder was mixed evenly to obtain a homogeneous sample.

The extraction of PAEs from the flooring samples was conducted using a combination of ultrasonic extraction and solid–liquid extraction methods. Specifically, 0.1 g of powdered flooring sample was weighed into a clean 10 mL glass centrifuge tube and spiked with 100 μL of a mixed internal standard solution of PAEs (DMP-d_4_, DEP-d_4_, DIBP-d_4_, DBP-d_4_, DHxP-d_4_, BBP-d_4_, DCHP-d_4_, DEHP-d_4_, DOP-d_4_) at a concentration of 200 ng/mL. After equilibration for 2 h, 2 mL of Milli-Q water and 4 mL of hexane were added, and the mixture was ultrasonically extracted for 30 min. The sample was then extracted by shaking for 60 min, followed by centrifugation at 4000× *g* for 10 min. The resulting supernatant was transferred into another cleaned glass tube and kept for later experiments. Subsequently, 2.0 mL of methanol was added to the sample, vortex-mixed, and extracted for 1 h. Then, 4.0 mL of hexane was added, vortex-mixed again, and extracted for 1 h. After a second centrifugation at 4000 rpm for 10 min, the resulting supernatant was collected and combined with the first extract. The extract was slowly concentrated to 0.5 mL under high-purity nitrogen, transferred to a 1.5 mL injection vial, and stored in the dark at 4 °C until instrumental analysis.

Nine target PAEs in flooring samples were quantified using an Agilent Technologies 7890A Gas Chromatography system coupled with a 5975C Mass Spectrometer system (Agilent, Santa Clara, CA, USA). Detailed information on the instrumental analysis was described in our previous study [14]. A DB-5MS capillary column (30 m × 0.25 × 0.25 μm, Agilent, Santa Clara, CA, USA) was used to achieve chromatographic separation. The injection volume was 1 μL, and the injection port temperature was set to 290 °C. The column temperature was initially set at 150 °C, with helium as the carrier gas at a flow rate of 0.9 mL/min. The temperature program for the column oven was as follows: it was heated from 60 °C to 150 °C at a rate of 20 °C/min, held for 2 min, then heated from 150 °C to 290 °C at a rate of 8 °C/min, and held for 4 min. The ion source used was an electron impact ionization (EI) source, and data were collected in the selected ion monitoring (SIM) mode. The ion source and quadrupole temperatures were set to 230 °C and 150 °C, respectively. The retention times and scan ion information for the target PAEs are listed in Table S3.

### 2.5. Quality Assurance and Quality Control (QA/QC)

For each batch of 20 samples, two method blanks, two spiked blanks, and two pairs of matrix spike samples were processed. The method blank samples were prepared by following exactly the same procedures as the real samples but without adding any flooring material. Their purpose was to monitor background contamination introduced during sample handling, grinding, solvent extraction, or instrument analysis. Spiked blanks were prepared by adding known quantities of target PAEs into blank matrices (no flooring material), and processed through the full analytical procedure. Their purpose was to evaluate the recovery efficiency of the analytical method and potential matrix-independent losses during extraction or analysis. Matrix spike samples were prepared by spiking real flooring samples with known concentrations of target PAEs. This type of quality control was used to assess matrix effects and validate the accuracy and reproducibility of the method in the presence of sample-specific interferences. The recoveries of isotope internal standards in flooring samples were 77 ± 16%, 112 ± 16%, 118 ± 20%, 112 ± 13%, 109 ± 24%, 103 ± 32%, 120 ± 26%, 124 ± 30%, and 131 ± 57%, respectively. Trace concentrations of DMP (4.05 ng/g), DEP (0.851 ng/g), DIBP (9.58 ng/g), DBP (5.08 ng/g) and DEHP (3.40 ng/g) were detected in the procedural blanks. The concentrations measured in the procedural blanks were subtracted from the concentrations. The limit of detection (LOD) of DMP, DEP, DIBP, DBP, and DHxP was 2.5 ng/g, and the LOD of BBP, DEHP, DCHP, and DOP was 5.0 ng/g. The limit of quantification (LOQ) for PAEs in all samples was 10–20 ng/g. Concentrations below the LOQ were substituted with a value equal to LOD divided by the square root of 2 for the calculation.

### 2.6. Migration Rate of PAEs in Flooring

To quantify the migration behavior of PAEs in flooring materials over time, the migration rate (*f*_i_) was calculated based on the relative reduction in PAE concentration between two sampling time points. The calculation is defined by the following equation:

$$f_{i}=\frac{C_{1}-C_{2}}{C_{1}}$$

where *C*_1_ and *C*_2_ represent the concentrations of PAEs in flooring in 2022 and 2023, respectively (ng/g). A positive value of *f*_i_ indicates a net release of PAEs from the material into the surrounding environment, while a negative value suggests reabsorption or external contamination. This normalized approach accounts for the initial concentration differences between samples and allows for more consistent comparisons of migration behavior across different flooring types.

### 2.7. Annual Variation of PAEs in Flooring

Based on the concentrations of PAEs in different types of flooring and the results of the questionnaire survey on the use of building materials in Chinese households, the annual variation in the total amount of PAEs in flooring used in newly renovated homes in China was calculated using the following equation:

$$B={(C}_{1}-C_{2})\times w\times S\times f_{i}$$

where *B* is the annual variation in the total amount of PAEs in flooring in newly renovated Chinese households (ng); *C*_1_ and *C*_2_ represent the concentrations of PAEs in flooring in 2022 and 2023, respectively (ng/g); *w* is the mass of flooring per unit area (g/m^2^); *S* is the floor coverage area (m^2^); *f_i_* is the migration rate of PAEs in the flooring.

### 2.8. Statistical Analysis

Statistical analyses were performed using SPSS 24.0, while data visualization was conducted with Origin 2024 Pro (Student Edition). The Mann–Whitney U test and Kruskal–Wallis H test were employed to analyze differences between groups. Due to the limited sample size and the non-normal distribution of the data, Spearman’s rank correlation coefficients were used to analyze the relationships between variables. Univariate regression analysis was used to assess the correlations among groups. Variables with statistical significance were included in the multiple linear regression analysis. Multicollinearity among the factors was excluded, and regression coefficients were calculated to determine the associations among the factors. The statistical significance level was set at *p* < 0.05.

## 3. Results and Discussion

### 3.1. Use of Decoration Materials in Chinese Households

The results of the questionnaire survey on the use of decoration materials in newly renovated homes in China are shown in Table 1. The results showed that newly renovated homes in urban areas accounted for the majority of the surveyed households (62.8%), indicating that renovation activities are more prevalent in urban settings, possibly due to better economic conditions and housing turnover. Apartments are the most common housing type (61.3%), which is consistent with the high proportion of urban samples. Most households (62.6%) have a living area of less than 120 m^2^, and more than 70% of homes have no more than three rooms, suggesting that newly renovated homes in China are predominantly small to medium-sized. The highest proportion of households (50.4%) have an annual income of less than USD 30,000, and the lowest proportion (only 13.4%) have an annual income greater than USD 70,000, indicating that newly renovated homes in China are primarily occupied by middle- and low-income groups. The survey results on renovation conditions showed that the renovation budgets of newly renovated homes in China are relatively balanced, each accounting for approximately 23% to 27% of the sample, suggesting that renovation investment is fairly balanced among different income levels.

The most common flooring materials are solid wood flooring (38.7%), followed by tiles (26.7%), engineered flooring (19.0%), and laminate wood flooring (15.7%). Wall decorations are primarily dominated by paint coatings (43.4%), followed by wallpaper (26.4%) and tiles (17.9%). The most frequently used types of engineered wood in newly renovated homes are plywood and solid wood panels, with 85.9% and 80.3% of newly renovated homes using these materials, respectively. Regarding living habits, the majority of residents spend 8–10 h at home daily (37.2%), with 19.4% of respondents staying at home for more than 14 h per day. Additionally, newly renovated homes generally have a high frequency of ventilation, with 76.9% of households ventilating at least once a day, while only 23.1% ventilate less than once a day, indicating a generally good awareness of ventilation among residents. These behavioral factors have a significant impact on indoor pollutant exposure, especially for individuals who spend extended periods at home.

### 3.2. Concentrations and Compositions of PAEs in Flooring

Phthalates were widely detected in flooring samples, indicating the extensive use of these plasticizers in flooring materials. Among the nine target PAEs, DIBP, DBP, and DEHP were detected in 100% of the samples, while DMP and DEP also exhibited high detection frequencies (98%), suggesting that these compounds are commonly used as primary plasticizers in flooring products (Table 2). The total concentrations of nine PAEs (Σ_9_ PAEs) varied significantly across different samples, ranging from 1220 ng/g to 166,000 ng/g, with a median concentration of 14,100 ng/g. In terms of compositional profiles, DEHP was the predominant compound, with a median concentration of 4290 ng/g, accounting for 62.8% of the total PAE concentration (Figure S1). As one of the most widely used plasticizers in the Chinese market, DEHP is commonly added to PVC flooring, adhesives for engineered wood flooring, and surface-protective films [33,34]. Due to its stable molecular structure and low volatility, it often remains within the material and is released over an extended period [35]. Its high proportion in the composition reflected its dominant role in flooring formulations. These findings are supported by several previous studies. A Japanese study reported similar results, where the highest concentrations of DEHP were detected in floor and multi-surface dust samples [36]. Wang et al. also found DEHP to be the most abundant PAE in both flat materials and plastic toys, with a detection frequency of 100%, reinforcing the conclusion that DEHP is the dominant plasticizer in current use [37]. Xue et al. further confirmed the frequent occurrence of DEHP in PVC wallpapers and flooring based on samples collected in Beijing, China [38]. These consistent observations from various studies underline the pervasive use of DEHP in indoor materials.

Additionally, DBP and DIBP contributed significantly to the overall concentration levels, with median concentrations of 1740 ng/g and 845 ng/g, respectively, accounting for approximately 14.9% and 8.9% of the total PAE concentration in the flooring samples. In some samples, the concentration of DBP was comparable to that of DEHP. DBP and DIBP are often used as auxiliary plasticizers or substitutes for DEHP, and they are found in adhesives, composite board cores, and base sealing layers. Their notable presence suggests the widespread use of phthalate-containing adhesive systems or binder layers in flooring production. In contrast, although DMP and DEP had high detection frequencies (98%), their concentration contributions were minimal. Their median concentrations were 1120 ng/g and 99.8 ng/g, respectively, indicating that they are not major functional additives in flooring. Instead, they may originate from surface coatings or environmental contamination during storage and transport. Other PAEs such as BBP, DHxP, DCHP, and DOP were detected in only a few samples at low concentrations, suggesting limited application in current mainstream flooring products.

The concentrations and detection frequencies of PAEs in different types of flooring are summarized in Table 2. The Σ_9_ PAEs, including DMP, DEP, DIBP, DBP, DHxP, BBP, DCHP, DEHP, and DOP, exhibited notable differences across flooring types. The highest total concentration was observed in solid wood flooring, with values ranging from 2020–166,000 ng/g (median: 55,900 ng/g), followed by engineered flooring (4500–99,000 ng/g, median: 22,600 ng/g). In contrast, laminate flooring showed significantly lower concentrations, ranging from 1220 to 49,900 ng/g (median: 4000 ng/g; *p* < 0.05). Among the individual compounds, DEHP, DBP, DIBP, DMP, and DEP were the most frequently detected, with 100% detection rates in solid wood and engineered flooring, and 93–100% in laminate flooring. DEHP consistently exhibited the highest concentrations across all flooring types, with median values of 30,400 ng/g in solid wood, 14,700 ng/g in engineered wood, and 1950 ng/g in laminate flooring (*p* < 0.05). It accounted for 68.7%, 65.9%, and 53.9% of total Σ_9_ PAEs, respectively, indicating its dominant contribution to the overall PAE burden.

The proportions of DMP, DEP, DIBP, and DBP were each approximately 10% in most samples. However, DBP showed a relatively higher contribution in laminate flooring, accounting for up to 24% of Σ_9_ PAEs. This may be related to its widespread use in epoxy resins, cellulose esters, and specialized adhesives applied in laminate flooring production [39,40]. Interestingly, BBP was detected in 33% of engineered flooring samples, while it was absent in both solid wood and laminate flooring. This selective presence may be attributed to the use of BBP-containing adhesives in the glue-roller application of urea–formaldehyde resin during engineered wood manufacturing processes [41]. Although DHxP, DCHP, and DOP were rarely detected, their presence in a few samples suggests occasional use of certain plasticizer additives or recycled components.

The observed differences in PAE concentrations across flooring types may be attributed to variations in material composition, surface treatment, and manufacturing processes [42]. Solid wood flooring, despite its natural composition, often undergoes surface finishing treatments such as lacquering, oiling, or waxing, which may contain high levels of plasticizers, especially DEHP and DBP [43]. Additionally, waxes and varnishes used to enhance durability and aesthetics may significantly contribute to the overall PAE load. Engineered flooring typically consists of multiple bonded layers, including a surface veneer and a plywood or high-density fiberboard core, which are commonly adhered using resin-based glues. These adhesives, especially those incorporating urea–formaldehyde or polyvinyl acetate, may contain or be contaminated with PAEs, particularly BBP and DIBP. The variability in glue formulations and adhesive application techniques (e.g., glue-rolling or hot-pressing) could partly explain the wider concentration range observed. In contrast, laminate flooring is often constructed with a melamine resin-impregnated paper layer and a high-density fiberboard core, both of which tend to contain fewer plasticizers. The heat and pressure used during lamination may also reduce the residual PAE content by promoting volatilization or thermal degradation. Moreover, laminate flooring often features a sealed top layer, which can act as a barrier limiting PAE release and accumulation.

### 3.3. Migration of PAEs in Flooring

**Figure 1:**
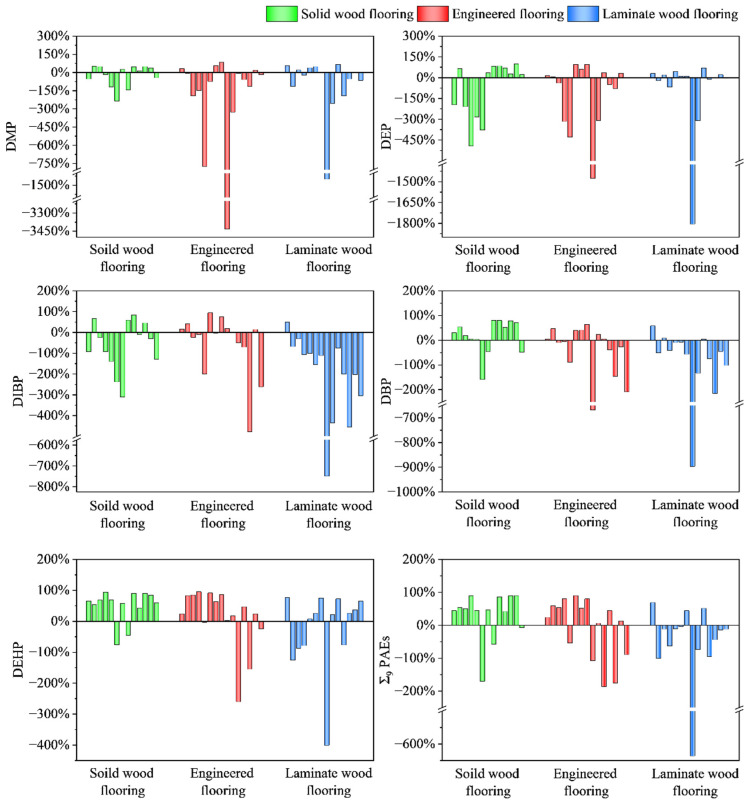
The concentration change rates of PAEs in different types of flooring. Positive values indicate the net emission (release) of PAEs from the flooring material, while negative values suggest net accumulation, potentially due to adsorption or retention from the surrounding environment.

The migration of PAEs in different types of flooring is shown in Figure 1. The results were inferred based on the comparison of PAE concentrations measured in flooring samples on day 1 and day 365 under natural indoor exposure conditions. A decrease in concentration was interpreted as a potential release of PAEs from the material, while an increase was considered indicative of uptake from the surrounding environment. In solid wood flooring, the release tendency of PAEs was generally prevalent, with 77% and 85% of the samples exhibiting a net decrease in DBP and DEHP concentrations, respectively. The corresponding concentration change rates were 30% and 65% (median value), indicating that most solid wood flooring commonly acts as a source of PAEs under typical indoor conditions. This trend may be related to the relatively loose structure and higher residual PAE content, making it more likely to release PAEs during the early stages of use. In contrast, DIBP showed a median concentration change rate of −30% in solid wood flooring, indicating an increase in concentration in some samples, which could be influenced by the background concentration in the indoor environment and its own diffusion behavior.

In laminated flooring, a more pronounced trend of the net accumulation of PAEs was observed. Specifically, 93%, 79%, and 57% of the samples showed increasing concentrations of DIBP, DBP, and DMP, respectively, with all median change rates being negative: −132% for DIBP, −48% for DBP, and −11% for DMP. These results suggest that laminated flooring may act as a “sink” for airborne PAEs under certain conditions, accumulating pollutants over time. This phenomenon may be influenced by the chemical composition, adsorption capacity, or sealing characteristics of laminated flooring. For instance, the melamine-impregnated paper commonly used in the surface layer may interact with PAEs via adsorption or molecular bonding, leading to accumulation on the surface or at internal interfaces. Notably, concentration change rates showed large variability—for example, DMP ranged from −1450% to 65%—indicating significant heterogeneity across product types. These differences warrant further investigation through detailed structural and compositional analysis.

Engineered flooring exhibited a dual migration behavior, with some samples showing increasing and others decreasing concentrations. For DIBP and DBP, 47% of the samples exhibited a net concentration increase and 53% a decrease, with median change rates of −3% and −5%, respectively. These values were close to zero, but with large variability (DIBP: −478% to 94%; DBP: −668% to 64%), suggesting inconsistent migration tendencies. At the total concentration level, the median change rate was 24%, with a range from −186% to 90%, further illustrating the complexity of behavior. Engineered flooring typically consists of multiple layers of various engineered woods and adhesives, leading to a complex internal structure and heterogeneous formulation. This heterogeneity likely accounts for the variation in PAE migration behavior. In addition, observed fluctuations may result from residual unreacted plasticizers or surface restructuring during environmental exposure, affecting migration direction and intensity.

The different migration characteristics of PAEs in different types of flooring suggested that the selection of flooring materials can have a significant impact on the indoor PAE pollution burden. For solid wood flooring, due to its relatively rough surface structure and the fact that PAE release is often associated with material aging and usage conditions, it may become an important source of PAE release in indoor environments, thereby affecting indoor air quality and human health. In contrast, laminated flooring, due to its relatively sealed structure and chemical properties, may absorb more PAEs from the indoor environment, thereby reducing the PAE concentration in the air to some extent, especially in high-pollution environments. The behavior of engineered flooring is more complex, as its multi-layer composite structure and interactions between different materials can lead to both the release and absorption of PAEs, increasing the uncertainty in its actual usage.

### 3.4. Factors Affecting the Migration of PAEs in Flooring

The migration of PAEs from flooring materials is influenced by multiple intrinsic factors, including initial PAE concentration, material type, and density [44,45,46,47]. To investigate the effects of these factors on PAE migration in flooring, variables with statistical significance from univariate regression analysis were incorporated into a multiple linear regression model, with PAE migration as the dependent variable (Table 3). The results showed that the initial concentration and density were significantly positively correlated with the migration of DEHP and Σ_9_ PAEs, while the material type was significantly positively correlated with DEHP migration (*p* < 0.05). Among all the variables, initial concentration had the most significant effect on PAE migration.

The initial concentrations of PAEs were positively associated with both DEHP and Σ_9_ PAE concentrations (*p* < 0.05), suggesting that higher initial concentrations promote greater outward migration. This result is consistent with Fick’s law of diffusion, which states that a higher concentration gradient between the material interior and the surrounding environment increases the diffusion flux. PAEs in building materials such as DEHP are known to follow the Fickian diffusion kinetic approximation model [48,49]. Thus, elevated initial concentrations result in stronger chemical potential gradients, accelerating migration to surrounding media (e.g., air or adjacent surfaces). Additionally, high initial levels can prolong the emission duration and increase the cumulative release over time.

Material type also showed a significant effect on DEHP migration. Flooring made of solid wood exhibited higher DEHP migration than engineered wood (*p* < 0.05). This may be attributed to the porous structure of solid wood, which facilitates molecular diffusion from the interior to the surface. While solid wood is capable of absorbing airborne SVOCs due to its fibrous matrix [50], the same structural features—such as interconnected pores and capillaries—may also support inward-to-outward transport of PAEs under strong internal concentration gradients. Furthermore, changes in the moisture content of wood can modulate the pore structure and diffusivity of solid wood, indirectly influencing the emission behavior of embedded PAEs [51,52].

Material density demonstrated a significant positive correlation with DEHP and Σ_9_PAE migration (*p* < 0.05). Generally, flooring materials with higher density have reduced free volume and a more compact internal structure. In high-density matrices, polymer chains or fibers are tightly packed, which can restrict the mobility of PAE molecules and compress their diffusion channels. Lower porosity and more tortuous diffusion paths also further hinder the transport of molecules to the surface. However, this trend was not observed in the present study. One possible explanation is that denser materials possess a higher polymer content and a more compact internal structure, which can trap larger amounts of PAEs within the matrix. While increased density may reduce diffusivity due to lower porosity and higher tortuosity, the overall reservoir of PAEs available for migration is larger, leading to greater cumulative release over time [53]. Thus, the positive relationship between density and PAE migration is likely due to a higher initial PAE burden and storage capacity in denser materials.

### 3.5. Estimation of the Environmental Burden of PAEs from Flooring

To assess the impact of flooring on the indoor pollution of PAEs in Chinese households, the potential environmental burden of PAEs introduced by flooring materials in newly renovated households was estimated. The estimated environmental burden of PAEs resulting from flooring in newly renovated Chinese households ranged from −3.63 × 10^9^ ng to 3.45 × 10^11^ ng, with a median value of 1.23 × 10^10^ ng (Table S4). The presence of negative values in the dataset is attributed to certain flooring types exhibiting net adsorption behavior for some PAEs, such as DMP, DBP, and DIBP, where the amount adsorbed exceeds the amount released, resulting in an overall “net adsorption effect”. Among the PAEs studied, DEHP accounted for the highest environmental burden, ranging from 4.23 × 10^7^ ng to 2.39 × 10^11^ ng, with a median value of 1.19 × 10^10^ ng, significantly exceeding the levels of other PAEs (*p* < 0.05). This finding indicated that DEHP plays a dominant role in flooring-related indoor PAE pollution.

**Figure 2:**
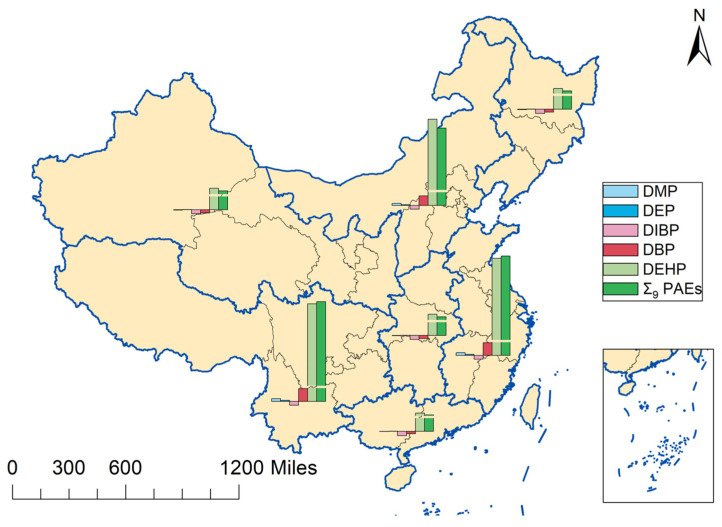
Environmental burdens of PAEs from flooring materials in newly renovated households.

Significant regional differences in flooring-related PAE burdens were observed across different geographical regions of China (*p* < 0.05) (Figure 2). The highest median values were found in households in East China and Southwest China. The estimated range for East China was −2.72 × 10^9^ ng to 1.23 × 10^11^ ng (median: 1.96 × 10^10^ ng), while that for Southwest China was −8.78 × 10^8^ ng to 3.68 × 10^10^ ng (median: 1.96 × 10^10^ ng). In contrast, households in South China exhibited the lowest total PAE burden, ranging from −9.08 × 10^8^ ng to 6.62 × 10^10^ ng, with a median value of 1.19 × 10^9^ ng. Further analysis revealed that the DEHP burden in East China was significantly higher than that in Northeast, Central, Northwest, and South China (*p* < 0.05). These regional differences may be attributed to variations in the types of flooring materials used in newly renovated homes, living areas, renovation habits, and economic conditions.

To further investigate the determinants of PAE environmental burdens arising from flooring, we analyzed the influence of multiple variables, including flooring type, housing type, living area, number of rooms, household income, and renovation budget (Tables S5–S10). The results showed that flooring material type was the most critical factor influencing the level of PAEs. The median environmental burdens associated with different flooring types were as follows: solid wood flooring (2.94 × 10^10^ ng) > engineered wood flooring (1.59 × 10^9^ ng) > laminate flooring (−7.26 × 10^8^ ng), with all group differences being statistically significant (*p* < 0.05). Notably, households with laminate flooring exhibited a negative net PAE burden, indicating that during use, DMP, DBP, and DIBP were primarily adsorbed, while only DEP and DEHP were released, resulting in an overall net adsorption effect. It is important to clarify that no adsorption experiments were conducted in this study. The observed negative net PAE burden in households with laminate flooring reflects a decrease in the internal concentrations of certain PAEs, which may suggest limited reuptake or environmental interaction processes under real-world conditions. This observation does not imply that laminate flooring actively adsorbs PAEs or functions as a pollution control material. Rather, it may indicate a complex interplay between flooring materials and their indoor environment over time.

Socioeconomic factors, including living area, number of rooms, household income, and renovation budget, also significantly influenced PAE burdens. Households with a living area ≥ 137 m^2^ had a median PAE burden of 3.44 × 10^10^ ng, significantly higher than those with areas of 86–120 m^2^ (2.21 × 10^10^ ng) and <85 m^2^ (4.91 × 10^9^ ng) (*p* < 0.05). Similarly, homes with more rooms exhibited higher PAE burdens. Households with more than 7 rooms or 4–6 rooms had DEHP burdens of 2.15 × 10^10^ ng and 1.67 × 10^10^ ng, respectively, significantly higher than households with 3 or fewer rooms (1.23 × 10^10^ ng) (*p* < 0.05). This pattern likely reflects that households with more rooms typically require a larger quantity of flooring materials, further increasing the overall PAE burden. Additionally, households with renovation budgets exceeding 205 USD/m^2^ had significantly higher DIBP burdens than those spending less than 125 USD/m^2^ (*p* < 0.05). This trend suggested that higher-income households tend to engage in larger-scale or more refined interior renovations, involving greater amounts and a wider variety of flooring. As renovation budgets increase, so do the intensity of flooring material use and the complexity of construction, which in turn amplifies the indoor environmental burden of PAEs.

## 4. Conclusions

This study investigated the occurrence and migration behavior of PAEs in different types of flooring, revealing the migration patterns of PAEs across various materials. PAEs are widespread in flooring materials. Different types of flooring exhibited distinct migration behaviors. Specifically, solid wood flooring tended to continuously release PAEs as an indoor source, whereas engineered wood flooring showed both emission and absorption behaviors, while laminate flooring notably absorbed PAEs from the surrounding environment. The significant regional differences in the environmental burden of PAEs from flooring in newly renovated households across China further highlight the impact of regional flooring material choices and renovation practices. In practical applications, it is recommended to select flooring materials with lower initial PAE concentrations and avoid those with high PAE content to reduce indoor PAE concentrations. Additionally, using laminated flooring or specially treated engineered wood flooring can help to minimize exposure. However, the actual effectiveness may vary depending on specific product formulations and environmental conditions, and further studies are needed to confirm long-term performance.

There are several limitations in our study. First, the flooring samples analyzed were limited to common materials used in Chinese households, which may not fully represent the migration behavior of PAEs in all types of flooring. Second, the long-term migration behavior of PAEs (>1 year) was not investigated in this study. PAE migration in flooring materials should be tracked over extended periods to better understand the effects of material aging on PAE release in the future. Third, the experiments in this study were conducted under constant temperature and humidity conditions, and the influence of indoor temperature and humidity on long-term PAE migration was not considered. It should be noted that the primary goal of this study was not to quantify the theoretical maximum release of PAEs in an isolated environment, but rather to simulate and assess the actual behavior of PAEs within flooring materials under realistic indoor conditions. In real indoor settings, PAEs can undergo both emission and reabsorption processes influenced by background airborne levels. Thus, instead of avoiding airborne PAEs entirely through airtight containment, we intentionally adopted a naturally ventilated setup to capture the net migration behavior of PAEs—whether outward or inward—reflecting what might occur in lived environments. Temperature is one of the main factors affecting PAE migration, and future studies should incorporate variable environmental conditions to better simulate real indoor environments and assess their impact on PAE release over time. Additionally, this study focused solely on the quantification of residual PAEs in flooring materials. Airborne concentrations of PAEs were not measured, which limits the direct assessment of emission rates and potential human exposure via inhalation. Future studies should incorporate air sampling and dynamic environmental conditions to provide a more comprehensive emission profile of SVOCs from indoor materials.

## Figures and Tables

**Table 1:** Questionnaire survey results on the use of decoration materials in newly renovated homes in China.

Category	Information				
Urban/rural attribute	Urban	Rural			
	424 (62.8%)	251 (37.2%)			
Housing type	Villa	Apartment	Self-built house		
	80 (11.9%)	414 (61.3%)	181 (26.8%)		
Living area ^a^ (m^2^)	≤85 m^2^	86–120 m^2^	121–136 m^2^	≥137 m^2^	
	171 (25.3%)	252 (37.3%)	84 (12.4%)	168 (24.9%)	
Room number	≤3	4–6	≥7		
	483 (71.6%)	176 (26.1%)	16 (2.3%)		
Household income (USD)	<30,000	30,000–70,000	70,000–150,000	>150,000	
	340 (50.4%)	245 (36.3%)	72 (10.7%)	18 (2.7%)	
Renovation budget (USD/m^2^)	<125	125–205	205–320	>320	
	161 (23.9%)	183 (27.1%)	174 (25.8%)	157 (23.3%)	
Flooring material	Solid wood flooring	Engineered flooring	Laminate wood flooring	Tile	
	261 (38.7%)	128 (19.0%)	106 (15.7%)	180 (26.7%)	
Wall covering	Wallpaper	Wall cloth	Tile	Paint	
	178 (26.4%)	83 (12.3%)	121 (17.9%)	293 (43.4%)	
Wooden furniture type ^b^	Tables and Chairs	Wardrobe	Kitchen	Bed	Door
	462 (68.45%)	650 (96.6%)	640 (94.8%)	580 (85.9%)	570 (84.4%)
Engineered wood material	Solid wood	Plywood	Particle board	Fiberboard	Other
	542 (80.3%)	580 (85.9%)	472 (69.9%)	380 (56.3%)	105 (16.0%)
Indoor time (hour)	<8	8–10	11–13	14–24	
	90 (13.3%)	251 (37.2%)	203 (30.1%)	131 (19.4%)	
Ventilation frequency (times/day)	2–3	1	0.5	Almost never	Never
	256 (37.9%)	263 (39.0%)	63 (9.3%)	61 (9.0%)	32 (4.7%)

The table presents the questionnaire results using a grouped format, with each category listed alongside its respective options and their corresponding frequencies and percentages. Rows within each category represent different response options. ^a^ The classification of living area and renovation budget is based on the quartiles derived from the survey data. ^b^ As multiple types of wooden furniture and engineered wood materials were often used in a single household, the total counts exceeded the number of surveyed households (*n* = 675).

**Table 2:** Concentrations (ng/g) of PAEs in different types of flooring.

Value	DMP	DEP	DIBP	DBP	DHxP	BBP	DCHP	DEHP	DOP	Σ_9_ PAEs ^a^
Total (*n* = 42)										
DF ^b^	98%	98%	100%	100%	2%	14%	0%	100%	0%	
Min ^c^	nd ^f^	nd	169	295	nd	nd	nd	606	nd	1220
Median ^d^	1120	99.8	845	1740	nd	nd	nd	4290	nd	14,100
Max ^e^	18,600	20,400	22,900	13,000	1010	801	nd	160,000	nd	166,000
Solid wood flooring (*n* = 13)										
DF	100%	100%	100%	100%	0%	8%	0%	100%	0%	100%
Min	188	6	169	312	nd	nd	nd	1350	nd	2020
Median	2610	129	1103	2640	nd	nd	nd	30,400	nd	55,900
Max	18,600	20,400	22,800	13,000	nd	801	nd	160,000	nd	166,000
Engineered flooring (*n* = 15)										
DF	100%	100%	100%	100%	7%	33%	0%	100%	0%	100%
Min	172	18.2	426	892	nd	nd	nd	1090	nd	4500
Median	1080	153	1280	1680	nd	nd	nd	14,700	nd	22,600
Max	11,100	1360	14,300	5630	1010	56.4	nd	95,500	nd	99,000
Laminate wood flooring (*n* = 14)										
DF	93%	93%	100%	100%	0%	0%	0%	100%	0%	100%
Min	nd	nd	171	295	nd	nd	nd	606	nd	1220
Median	229	40	452	936	nd	nd	nd	1950	nd	4000
Max	3880	276	4560	8980	nd	nd	nd	32,200	nd	49,900

All concentrations are expressed in nanograms per gram (ng/g). ^a^ Σ_9_ PAEs refers to the total concentration of nine PAEs, including DMP, DEP, DIBP, DBP, DHxP, BBP, DCHP, DEHP, and DOP. ^b^ DF stands for detection frequency, indicating the percentage of samples in which the compound was detected. ^c^ Min represents the minimum detected concentration. ^d^ Median represents the median concentration used to describe the central tendency of the data. ^e^ Max represents the maximum detected concentration. ^f^ nd denotes not detected, indicating concentrations below the detection limit.

**Table 3:** Multiple linear regression analysis of factors influencing PAE migration from flooring.

Variable	β (95% CI) ^a^	*p*-Value
DEHP		
Initial concentration	1.005 (0.641~0.897)	0.000
Density	0.301 (21,500~113,000)	0.007
Solid wood flooring	0.235 (1200~33,200)	0.042
Laminate wood flooring	−0.075 (−20,100~−9370)	0.481
Σ_9_ PAEs		
Initial concentration	0.917 (0.614~1.02)	0.000
Density	0.332 (−14,500~158,000)	0.024
Solid wood flooring	0.220 (6410~43,900)	0.153
Laminate wood flooring	−0.051 (−27,300~18,900)	0.721

^a^ β represents regression coefficient; 95% CI represents 95% confidence interval.

## Data Availability

Data are contained within the article.

## Supplementary Materials

The following supporting information can be downloaded at: https://www.mdpi.com/article/10.3390/toxics13070517/s1. Table S1: Questionnaire on the use of decoration materials in newly renovated homes in China; Table S2: Information on flooring samples; Table S3: Information on retention time and quantitative and qualitative ions of target compounds; Table S4: Potential environmental burden of PAEs resulting from flooring in newly renovated Chinese households; Table S5: Estimated environmental burdens of PAEs in newly renovated households from different types of flooring (ng); Table S6: Estimated environmental burdens of PAEs from flooring in different housing types (ng); Table S7: Estimated environmental burdens of PAEs from flooring in households with different living areas (ng); Table S8: Estimated environmental burdens of PAEs from flooring in households with different room numbers (ng); Table S9: Estimated environmental burdens of PAEs from flooring in households with different renovation budgets (ng); Table S10: Estimated environmental burdens of PAEs from flooring in households with different total incomes (ng); Figure S1: Concentration compositions of PAEs in flooring.
