# Supplementary material for: Occurrence, Migration Behavior, and Environmental Burden of Phthalate Esters in Flooring Materials Used in Newly Renovated Chinese Homes
Source: Toxics. 2025 Jun 20;13(7):517. doi: 10.3390/toxics13070517 (PMC12299772; doi:10.3390/toxics13070517)
Supplement: Supplementary file 1 [file toxics-13-00517-s001.zip › toxics-3660113-supplementary.pdf]

Supplementary Material

Occurrence, Migration Behavior, and Environmental Burden of Phthalate Esters in Flooring Materials Used in Newly Renovated Chinese Homes

Ying Zhang [1], Li-Bo Chen [1], Hao-Yang Shen [1], Zi-Chao Wu [1], Ning-Zheng Zhu [2], Chong-Jing Gao [1,*] and Ying Guo [3]

[1] Zhejiang Provincial Top Discipline of Biological Engineering (Level A), Zhejiang Wanli University, Ningbo 315100, China; z_yingzy@163.com (Y.Z.); lbchen0107@163.com (L.-B.C.); shenhaoy2023@163.com (H.-Y.S.); wzc4811@163.com (Z.-C.W.)
[2] State Key Laboratory of Pollution Control and Resources Reuse, College of Environmental Science and Engineering, Tongji University, Shanghai 200092, China; zhuningzheng1984@163.com
[3] Guangdong Key Laboratory of Environmental Pollution and Health, College of Environment and Climate, Jinan University, Guangzhou 510632, China; yingguo2004@jnu.edu.cn
* Correspondence: gcj352535@163.com; Tel.: +86-0574-88222679

NO. OF TABLES: 10

NO. OF FIGURES: 1

NO. OF PAGES: 12

Table S1 Questionnaire on the use of decoration materials in newly renovated homes in China.

Question
1. What is your gender?
A. Male   B. Female
2. What is your age?
A. <18   B. 19-24   C. 25-40   D. 40-55   E. >55
3. What is your height (cm)? (Fill in the blank)
4. What is your weight (kg)? (Fill in the blank)
5. What is the location of your current residence?
A. Urban   B. Rural
6. What is the type of your residence?
A. Villa   B. Apartment   C. Self-built house
7. What is the living area of your house (m^2)? (Fill in the blank)
8. What is the number of permanent residents in your household? (Fill in the blank)
9. How many hours do you spend at home on average per day (hour)?
A. <8   B. 8-10   C. 11-13   D. 14-24
10. What is your household's total annual income? (Fill in the blank)
11. How much did you spend on home renovation? (Fill in the blank)
12. Which types of wooden furniture are present in your residence? (multiple choice)
A. Tables & Chair   B. Wardrobe   C. Kitchen   D. Bed   E. Door
13. What type of flooring material is used in your home?
A. Solid wood flooring   B. Engineered flooring
C. Laminate flooring   D. Tile
14. What type of wall decoration is used in your home?
A. Wall paper   B. Wall cloth   C. Tile   D. Paint
15. How often do you ventilate your indoor space (times/day)?
A. 2-3   B. 1   C. 0.5   D. Almost never   E. Never
16. Which of indoor pollutants are you aware of in decoration materials? (multiple choice)
A. Formaldehyde   B. Total Volatile Organic Compounds (TVOC)
C. Plasticizer (phthalate)   D. Benzene homologues (toluene, xylene, etc.)
E. Heavy metals (lead, mercury, etc.)   F. Others
17. During the renovation process, did you specifically choose materials with low pollutants?
A. Yes   B. No

Table S2 Information of flooring samples.

Sample	Type	Brand	Sample origin
Sample 1	Laminate wood flooring	Brand 1	Jiangsu Province
Sample 2	Engineered wood flooring	Brand 2	Zhejiang Province
Sample 3	Solid wood flooring	Brand 3	Guangdong Province
Sample 4	Solid wood flooring	Brand 2	Zhejiang Province
Sample 5	Laminate wood flooring	Brand 4	Jiangsu Province
Sample 6	Solid wood flooring	Brand 5	Guangdong Province
Sample 7	Solid wood flooring	Brand 3	Guangdong Province
Sample 8	Solid wood flooring	Brand 6	Zhejiang Province
Sample 9	Solid wood flooring	Brand 7	Zhejiang Province
Sample 10	Engineered wood flooring	Brand 5	Guangdong Province
Sample 11	Engineered wood flooring	Brand 7	Zhejiang Province
Sample 12	Laminate wood flooring	Brand 7	Zhejiang Province
Sample 13	Laminate wood flooring	Brand 8	Guangdong Province
Sample 14	Laminate wood flooring	Brand 9	Jiangsu Province
Sample 15	Solid wood flooring	Brand 9	Jiangsu Province
Sample 16	Engineered wood flooring	Brand 9	Jiangsu Province
Sample 17	Solid wood flooring	Brand 10	Guangdong Province
Sample 18	Solid wood flooring	Brand 11	Zhejiang Province
Sample 19	Engineered wood flooring	Brand 12	Guangdong Province
Sample 20	Laminate wood flooring	Brand 12	Guangdong Province
Sample 21	Laminate wood flooring	Brand 13	Jiangsu Province
Sample 22	Engineered wood flooring	Brand 13	Jiangsu Province
Sample 23	Engineered wood flooring	Brand 13	Jiangsu Province
Sample 24	Laminate wood flooring	Brand 14	Shandong Province
Sample 25	Engineered wood flooring	Brand 15	Guangdong Province
Sample 26	Laminate wood flooring	Brand 15	Guangdong Province
Sample 27	Laminate wood flooring	Brand 16	Jiangsu Province
Sample 28	Solid wood flooring	Brand 16	Jiangsu Province
Sample 29	Engineered wood flooring	Brand 16	Jiangsu Province
Sample 30	Engineered wood flooring	Brand 17	Jiangsu Province
Sample 31	Laminate wood flooring	Brand 17	Jiangsu Province
Sample 32	Engineered wood flooring	Brand 18	Zhejiang Province
Sample 33	Engineered wood flooring	Brand 18	Zhejiang Province
Sample 34	Solid wood flooring	Brand 19	Jiangsu Province
Sample 35	Engineered wood flooring	Brand 19	Jiangsu Province
Sample 36	Laminate wood flooring	Brand 19	Jiangsu Province
Sample 37	Engineered wood flooring	Brand 20	Jiangsu Province
Sample 38	Engineered wood flooring	Brand 20	Jiangsu Province
Sample 39	Solid wood flooring	Brand 20	Jiangsu Province
Sample 40	Laminate wood flooring	Brand 20	Jiangsu Province
Sample 41	Laminate wood flooring	Brand 21	Jiangsu Province
Sample 42	Solid wood flooring	Brand 21	Jiangsu Province

Table S3 Information on retention time, quantitative and qualitative ions of target compounds.

Target compound	Retention time (min)	Quantitative ion	Qualitative ion	Internal standard quantitative ion
DMP	9.069	163	77	167
DEP	11.045	149	177	153
DIBP	14.797	149	223	153
DBP	16.02	149	223	153
DHxP	20.399	149	251	153
BBP	20.517	149	91	153
DCHP	22.218	149	167	153
DEHP	22.342	149	167	153
DOP	24.198	149	279	283

Table S4 Potential environmental burden of PAEs resulting from flooring in newly renovated Chinese households (ng).

Compound	Min	Median	Max
DMP	-360.79	6.52×10^{7}	1.30×10^{9}
DEP	1.81×10^{5}	2.47×10^{7}	4.94×10^{8}
DIBP	-365.15	-166.32	-450.5
DBP	-203.83	2.98×10^{8}	5.96×10^{9}
DHxP	0.00	0.00	0.00
BBP	0.00	0.00	0.00
DCHP	0.00	0.00	0.00
DEHP	$4.23\text{E}\times10^{7}$	1.19×10^{10}	2.39×10^{11}
DOP	0.00	0.00	0.00
S_9PAEs	-395.67	1.23×10^{10}	2.45×10^{11}

Table S5 Estimated environmental burdens of PAEs in newly renovated households from different types of flooring (ng).

Type	Value	DMP	DEP	DIBP	DBP	DHxP	BBP	DCHP	DEHP	DOP	Σ_9PAEs
Solid wood	Min	2.61×10^{7}	9.88×10^{6}	-1.18×10^{9}	1.19×10^{8}	0.00	0.00	0.00	4.78×10^{9}	0.00	4.91×10^{9}
	Median	1.56×10^{8}	5.93×10^{7}	-1.42×10^{8}	7.15×10^{8}	0.00	0.00	0.00	2.87×10^{10}	0.00	2.94×10^{10}
	Max	1.30×10^{9}	4.94×10^{8}	-2.37×10^{7}	5.96×10^{9}	0.00	0.00	0.00	2.39×10^{11}	0.00	2.45×10^{11}
Engineered wood	Min	-3.31×10^{9}	2.34×10^{5}	-1.42×10^{8}	-9.39×10^{8}	0.00	0.00	0.00	5.30×10^{8}	0.00	3.98×10^{8}
	Median	-3.98×10^{8}	9.34×10^{5}	-1.70×10^{7}	-1.13×10^{8}	0.00	0.00	0.00	2.12×10^{9}	0.00	1.59×10^{9}
	Max	-9.95×10^{7}	7.78×10^{6}	-4.25×10^{6}	-2.82×10^{7}	0.00	0.00	0.00	1.76×10^{10}	0.00	1.33×10^{10}
Laminated wood	Min	-1.11×10^{8}	1.81×10^{5}	-3.35×10^{9}	-1.87×10^{9}	0.00	0.00	0.00	4.23×10^{7}	0.00	-3.63×10^{9}
	Median	-2.22×10^{7}	1.45×10^{6}	-6.70×10^{8}	-3.74×10^{8}	0.00	0.00	0.00	3.38×10^{8}	0.00	-7.26×10^{8}
	Max	-2.78×10^{6}	7.24×10^{6}	-8.38×10^{7}	-4.67×10^{7}	0.00	0.00	0.00	1.69×10^{9}	0.00	-9.08×10^{7}

Table S6 Estimated environmental burdens of PAEs from flooring in different housing types (ng).

Housing type	Value	DMP	DEP	DIBP	DBP	DHxP	BBP	DCHP	DEHP	DOP	Σ_9PAEs
Villa	Min	-3.31×10^{9}	5.45×10^{5}	-2.51×10^{9}	-1.40×10^{9}	0.00	0.00	0.00	1.69×10^{8}	0.00	-2.72×10^{9}
	Median	8.47×10^{7}	3.21×10^{7}	-1.66×10^{8}	3.87×10^{8}	0.00	0.00	0.00	1.67×10^{10}	0.00	1.59×10^{10}
	Max	6.52×10^{8}	2.47×10^{8}	-9.92×10^{6}	2.98×10^{9}	0.00	0.00	0.00	1.19×10^{11}	0.00	1.23×10^{11}
Apartment	Min	-5.81×10^{8}	1.81×10^{5}	-3.35×10^{9}	-1.87×10^{9}	0.00	0.00	0.00	4.23×10^{7}	0.00	-3.63×10^{9}
	Median	6.52×10^{7}	2.47×10^{7}	-1.42×10^{8}	2.98×10^{8}	0.00	0.00	0.00	1.19×10^{10}	0.00	1.23×10^{10}
	Max	3.91×10^{8}	1.48×10^{8}	-4.25×10^{6}	1.79×10^{9}	0.00	0.00	0.00	7.16×10^{10}	0.00	7.36×10^{10}
Self-built house	Min	-6.64×10^{8}	4.67×10^{5}	-2.23×10^{9}	-1.25×10^{9}	0.00	0.00	0.00	1.41×10^{8}	0.00	-2.42×10^{9}
	Median	-9.27×10^{6}	4.83×10^{6}	-1.54×10^{8}	-5.64×10^{7}	0.00	0.00	0.00	3.53×10^{9}	0.00	2.65×10^{9}
	Max	1.30×10^{9}	4.94×10^{8}	-8.50×10^{6}	5.96×10^{9}	0.00	0.00	0.00	2.39×10^{11}	0.00	2.45×10^{11}

Table S7 Estimated environmental burdens of PAEs from flooring in household with different living areas (ng).

Living area (m^2)	Value	DMP	DEP	DIBP	DBP	DHxP	BBP	DCHP	DEHP	DOP	Σ_9PAEs
<85	Min	-2.82×10^8	1.81×10^5	-4.63×10^8	-2.59×10^8	0.00	0.00	0.00	4.23×10^7	0.00	-5.02×10^8
	Median	2.61×10^7	9.88×10^6	-9.46×10^7	1.19×10^8	0.00	0.00	0.00	4.78×10^9	0.00	4.91×10^9
	Max	1.11×10^8	4.20×10^7	-4.25×10^6	5.07×10^8	0.00	0.00	0.00	2.03×10^{10}	0.00	2.09×10^{10}
86-120	Min	-3.98×10^8	7.01×10^5	-6.70×10^8	-3.74×10^8	0.00	0.00	0.00	2.46×10^8	0.00	-7.26×10^8
	Median	1.17×10^8	4.45×10^7	-1.36×10^8	5.37×10^8	0.00	0.00	0.00	2.15×10^{10}	0.00	2.21×10^{10}
	Max	1.56×10^8	5.93×10^7	-1.28×10^7	7.15×10^8	0.00	0.00	0.00	2.87×10^{10}	0.00	2.94×10^{10}
121-136	Min	-4.51×10^8	1.00×10^6	-7.59×10^8	-4.24×10^8	0.00	0.00	0.00	3.47×10^8	0.00	-8.23×10^8
	Median	-2.28×10^7	1.61×10^6	-1.54×10^8	-1.22×10^8	0.00	0.00	0.00	2.39×10^9	0.00	1.80×10^9
	Max	1.77×10^8	6.72×10^7	-1.83×10^7	8.11×10^8	0.00	0.00	0.00	3.25×10^{10}	0.00	3.34×10^{10}
>137	Min	-3.31×10^9	1.09×10^6	-3.35×10^9	-1.87×10^9	0.00	0.00	0.00	3.95×10^8	0.00	-3.63×10^9
	Median	1.82×10^8	6.92×10^7	-2.22×10^8	8.35×10^8	0.00	0.00	0.00	3.34×10^{10}	0.00	3.44×10^{10}
	Max	1.30×10^9	4.94×10^8	-1.98×10^7	5.96×10^9	0.00	0.00	0.00	2.39×10^{11}	0.00	2.45×10^{11}

Table S8 Estimated environmental burdens of PAEs from flooring in household with different room numbers (ng).

Room number	Value	DMP	DEP	DIBP	DBP	DHxP	BBP	DCHP	DEHP	DOP	Σ_9PAEs
	Min	-6.64×10^{8}	1.81×10^{5}	-8.93×10^{8}	-4.98×10^{8}	0.00	0.00	0.00	4.23×10^{7}	0.00	-9.68×10^{8}
<3	Median	6.52×10^{7}	2.47×10^{7}	-1.42×10^{8}	2.98×10^{8}	0.00	0.00	0.00	1.19×10^{10}	0.00	1.23×10^{10}
	Max	3.91×10^{8}	1.48×10^{8}	-4.25×10^{6}	1.79×10^{9}	0.00	0.00	0.00	7.16×10^{10}	0.00	7.36×10^{10}
	Min	-1.69×10^{9}	3.62×10^{5}	-3.35×10^{9}	-1.87×10^{9}	0.00	0.00	0.00	8.46×10^{7}	0.00	-3.63×10^{9}
4-6	Median	9.12×10^{7}	3.46×10^{7}	-1.75×10^{8}	4.17×10^{8}	0.00	0.00	0.00	1.67×10^{10}	0.00	1.72×10^{10}
	Max	6.52×10^{8}	2.47×10^{8}	-8.50×10^{6}	2.98×10^{9}	0.00	0.00	0.00	1.19×10^{11}	0.00	1.23×10^{11}
	Min	-3.31×10^{9}	5.43×10^{6}	-2.51×10^{9}	-1.40×10^{9}	0.00	0.00	0.00	1.27×10^{9}	0.00	-2.72×10^{9}
>7	Median	1.17×10^{8}	4.45×10^{7}	-1.48×10^{8}	5.37×10^{8}	0.00	0.00	0.00	2.15×10^{10}	0.00	2.21×10^{10}
	Max	1.30×10^{9}	4.94×10^{8}	-5.91×10^{7}	5.96×10^{9}	0.00	0.00	0.00	2.39×10^{11}	0.00	2.45×10^{11}

Table S9 Estimated environmental burdens of PAEs from flooring in household with different renovation budget (ng).

Renovation budget (USD/m^2)	Value	DMP	DEP	DIBP	DBP	DHxP	BBP	DCHP	DEHP	DOP	Σ_9PAEs
	Min	-6.64×10^{8}	5.45×10^{5}	-3.35×10^{9}	-1.87×10^{9}	0.00	0.00	0.00	1.41×10^{8}	0.00	-3.63×10^{9}
< 125	Median	-1.11×10^{7}	4.02×10^{6}	-1.66×10^{8}	-9.12×10^{7}	0.00	0.00	0.00	2.65×10^{9}	0.00	1.99×10^{9}
	Max	1.04×10^{9}	3.95×10^{8}	-9.92×10^{6}	4.77×10^{9}	0.00	0.00	0.00	1.91×10^{11}	0.00	1.96×10^{11}
	Min	-1.69×10^{9}	4.67×10^{5}	-1.51×10^{9}	-8.41×10^{8}	0.00	0.00	0.00	1.41×10^{8}	0.00	-1.63×10^{9}
125-205	Median	-1.02×10^{7}	3.62×10^{6}	-1.54×10^{8}	-5.64×10^{7}	0.00	0.00	0.00	5.83×10^{9}	0.00	4.38×10^{9}
	Max	1.30×10^{9}	4.94×10^{8}	-8.50×10^{6}	5.96×10^{9}	0.00	0.00	0.00	2.39×10^{11}	0.00	2.45×10^{11}
	Min	-3.31×10^{9}	4.67×10^{5}	-8.93×10^{8}	-9.39×10^{8}	0.00	0.00	0.00	1.41×10^{8}	0.00	-9.68×10^{8}
205-320	Median	9.12×10^{7}	3.46×10^{7}	-1.30×10^{8}	4.17×10^{8}	0.00	0.00	0.00	1.67×10^{10}	0.00	1.72×10^{10}
	Max	6.52×10^{8}	2.47×10^{8}	-8.50×10^{6}	2.98×10^{9}	0.00	0.00	0.00	1.19×10^{11}	0.00	1.23×10^{11}
	Min	-9.95×10^{8}	1.81×10^{5}	-2.51×10^{9}	-1.40×10^{9}	0.00	0.00	0.00	4.23×10^{7}	0.00	-2.72×10^{9}
> 320	Median	7.82×10^{7}	2.97×10^{7}	-1.54×10^{8}	3.58×10^{8}	0.00	0.00	0.00	1.43×10^{10}	0.00	1.47×10^{10}
	Max	3.91×10^{8}	1.48×10^{8}	-4.25×10^{6}	1.79×10^{9}	0.00	0.00	0.00	7.16×10^{10}	0.00	7.36×10^{10}

Table S10 Estimated environmental burdens of PAEs from flooring in household with different total income (ng).

Household income (USD)	Value	DMP	DEP	DIBP	DBP	DHxP	BBP	DCHP	DEHP	DOP	Σ_9PAEs
<30,000	Min	-2.65×10^{9}	1.81×10^{5}	-1.86×10^{9}	-1.04×10^{9}	0.00	0.00	0.00	4.23×10^{7}	0.00	-2.02×10^{9}
	Median	1.16×10^{7}	8.06×10^{6}	-1.42×10^{8}	3.63×10^{7}	0.00	0.00	0.00	5.97×10^{9}	0.00	6.13×10^{9}
	Max	3.91×10^{8}	1.48×10^{8}	-7.09×10^{6}	1.79×10^{9}	0.00	0.00	0.00	7.16×10^{10}	0.00	7.36×10^{10}
30,000-70,000	Min	-1.69×10^{9}	2.34×10^{5}	-3.35×10^{9}	-1.87×10^{9}	0.00	0.00	0.00	1.13×10^{8}	0.00	-3.63×10^{9}
	Median	9.12×10^{7}	3.46×10^{7}	-1.54×10^{8}	4.17×10^{8}	0.00	0.00	0.00	1.67×10^{10}	0.00	1.72×10^{10}
	Max	1.04×10^{9}	3.95×10^{8}	-4.25×10^{6}	4.77×10^{9}	0.00	0.00	0.00	1.91×10^{11}	0.00	1.96×10^{11}
70,000-150,000	Min	-5.81×10^{8}	5.45×10^{5}	-2.51×10^{9}	-1.40×10^{9}	0.00	0.00	0.00	2.51×10^{8}	0.00	-2.72×10^{9}
	Median	1.17×10^{8}	4.45×10^{7}	-1.67×10^{8}	5.37×10^{8}	0.00	0.00	0.00	2.15×10^{10}	0.00	2.21×10^{10}
	Max	3.91×10^{8}	1.48×10^{8}	-9.92×10^{6}	1.79×10^{9}	0.00	0.00	0.00	7.16×10^{10}	0.00	7.36×10^{10}
>150,000	Min	-3.31×10^{9}	6.23×10^{5}	-1.25×10^{9}	-9.39×10^{8}	0.00	0.00	0.00	1.69×10^{8}	0.00	-1.35×10^{9}
	Median	1.17×10^{8}	4.45×10^{7}	-3.15×10^{8}	5.37×10^{8}	0.00	0.00	0.00	2.43×10^{10}	0.00	2.26×10^{10}
	Max	1.30×10^{9}	4.94×10^{8}	-1.13×10^{7}	5.96×10^{9}	0.00	0.00	0.00	2.39×10^{11}	0.00	2.45×10^{11}

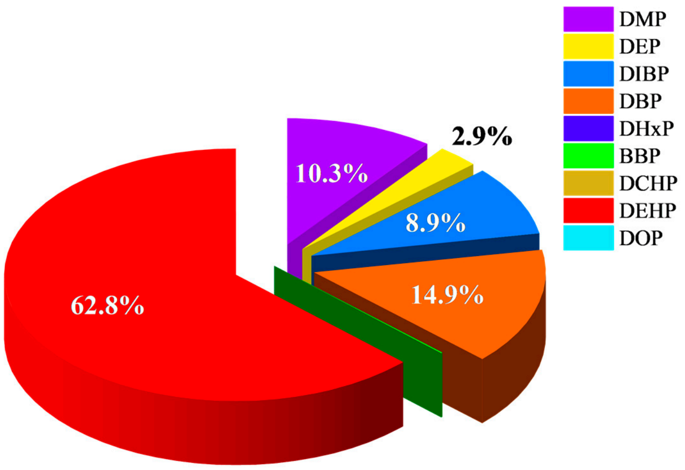

Figure S1 Concentration compositions of PAEs in flooring.
